# Testing With Intent in Mosaic Conditions: A Case-Based Review

**DOI:** 10.7759/cureus.49644

**Published:** 2023-11-29

**Authors:** Andrew J Kerwin, Ana L Lop, Kristyn Vicente, Tracey Weiler, Sajel L Kana

**Affiliations:** 1 Department of Genetics, Florida International University, Herbert Wertheim College of Medicine, Miami, USA; 2 Department of Genetics, New York Medical College, Valhalla, USA; 3 Department of Medical Education, Florida International University, Herbert Wertheim College of Medicine, Miami, USA; 4 Division of Clinical Genetics, Genomics, and Metabolism, Nicklaus Children's Hospital, Miami, USA

**Keywords:** tissue sampling, diagnostic odyssey, tuberous sclerosis complex, pallister-killian syndrome, genetic testing, mosaicism

## Abstract

Recent advancements in genetic testing have revealed cases of mosaicism, demonstrating the phenomenon may be more common than once thought. Broadly defined, mosaicism describes the presence of two genotypically different cell lineages within the same organism. This can arise from small mutations or errors in chromosome segregation, as early as in gametes, before or after fertilization. Mosaicism is directly responsible for many conditions that present in a wide range of tissues, with the presence of the mutation or genetic abnormality following a tissue-dependent pattern. This makes it possible for patients to test negative for a condition using a standard tissue sample while harboring the variant in a different tissue. Understanding the timing and mechanisms of mosaic conditions will aid in targeted testing that is more appropriate to identify a pathogenic variant. This targeted testing should reduce the length of a patient's diagnostic odyssey and provide a better understanding of the chances of passing on their variant to their offspring, thereby allowing for more accurate genetic counseling. We illustrate this phenomenon with two cases: one of Pallister-Killian syndrome and the other of tuberous sclerosis complex. Both patients had increased time to diagnosis because of difficulties in identifying genetic variants in tested tissues. Beyond just increased time to diagnosis, we illustrate that mosaic conditions can present as less severe and more variable than the germline condition and how specific germ layers may be affected by the variant. Knowing which germ layers may be affected by the variant can give clinicians a clue as to which tissues may need to be tested to yield the most accurate result.

## Introduction

One would expect each cell in a single individual to have the same genetic composition. As advancements in genetic testing have been made available, it has become evident that this is not always the case [1]. Mosaicism is a complex phenomenon widely encountered in medicine and is defined by the presence of more than one set of genetically unique cells in an individual originating from a single fertilized egg. This can arise at any point during the individual's development and as early on as gamete formation via nondisjunction events. Though meiotic nondisjunction has been traditionally associated with constitutional aneuploidies, it is now understood that post-fertilization chromosome rescue and mitotic recombination events may give rise to a mosaic phenotype in some patients. Errors after fertilization result from mistakes in gene duplication and mitotic division during embryonic development and thereafter. Regardless of the origin of the mutation, it has the ability to propagate over the course of many cell divisions (and perhaps additional errors in the DNA) to create a unique cell line. As a result, an individual will have more than one cell line, each with a unique genotype. That individual is said to have a mosaic genotype.

Mosaicism may be more common than originally thought and not necessarily involved in disease. In a study testing the frequency of mosaic mutations in 50,000 subjects, Laurie et al. found that detectable clonal mosaicism was found in >0.5% of young subjects but rapidly increased to 2-3% subjects after the age of 50 [2]. The mechanisms of these mutations are similar to any other mutations including, but not limited to, mitotic nondisjunction, single nucleotide variants (SNVs), or copy number variants (CNVs). Mutations missed by DNA repair mechanisms are passed down the cell line and can contribute to diseases, such as cancer, which go on to present in a mosaic pattern [3].

Oftentimes, mosaic conditions appear as milder phenotypes of a disease typically encountered in a constitutionally aneuploid form. One such condition is Down syndrome, of which an estimated 2-4% of diagnosed patients are considered mosaic Down syndrome (MDS). Due to the frequent absence of dysmorphic features, diagnosis of MDS patients is often a challenge; only 37.5% of MDS individuals are successfully diagnosed based on clinical features alone [4]. Another example is Klinefelter syndrome (KS), in which approximately 15-20% of patients are thought to have some form of mosaicism. In addition to milder physical characteristics, patients with mosaic KS are capable of reproducing at rates higher than their constitutionally aneuploid counterparts, who are typically infertile [5].

One important topic to consider in mosaic conditions is the mutant allele percentage (MAP) or the percentage of cells that display the mutation in a given tissue. MAP is likely correlated to both the phenotype and severity of mosaic diseases. Using MosaicBase, a comprehensive software designed to examine 383 cases of mosaicism reported in journal articles, Yang et al. found that mosaic patients with a higher MAP showed a statistically significant, more severe phenotype [6]. In a study of mosaicism in retinoblastoma patients, the mosaic frequency was found to correlate with the disease phenotype and the age of diagnosis [7]. Similarly, symptoms of expressive language delay and motor difficulties found in patients mosaic for Angelman syndrome, caused by post-fertilization imprinting errors, are more mild than those exhibited by patients affected by the constitutionally aneuploid form of the syndrome [8].

Here, we discuss two cases of genetic mosaicism: 1) Pallister-Killian syndrome (PKS) and 2) tuberous sclerosis complex (TSC). PKS, caused by pre-fertilization maternal meiotic nondisjunction, is characterized by the presence of an isochromosome, resulting in the tetrasomy of chromosome 12p [9]. The clinical presentation of PKS, although variable in severity, often includes intellectual disability, dysmorphology, seizures, and congenital malformations [10]. Despite its origin in gamete formation, all reported cases of PKS have been found in the mosaic form, perhaps caused by an inability of constitutional aneuploids to sustain life.

TSC is an autosomal dominant condition caused by mutations in either the TSC1 or TSC2 genes [11]. TSC is a multisystemic disease that presents with variable expressivity, including neurological disorders such as epilepsy, and the growth of benign tumors, including renal angiomyolipomas (AML) and facial angiofibromas [12]. Tyburczy et al. have demonstrated the prominence of mosaicism in cases of TSC [13]. Roughly 10-15% of the TSC patients in their study underwent initial genetic testing that did not identify mutations in either TSC1 or TSC2. In over half of these cases, the failure to initially identify the TSC1 or TSC2 variant was attributed to mosaicism. Documented cases of mosaic TSC have shown a lower median number of TSC manifestations and a less severe phenotype in patients with a lower MAP [13,14].

Both of these cases were previously presented as part of a poster at the American Medical Association (AMA) Research Challenge from October 21 to 23, 2021.

## Case presentation

Case 1: PKS

A one-year-old male from the Dominican Republic initially presented to a pediatric neurologist in Santo Domingo following concerns of regression in his speech and decreased muscle tone. A brain MRI was obtained, which failed to reveal any significant findings. The child began speech and occupational therapy in the Dominican Republic for three months, without progress.

At nearly two years of age, the patient was taken to Nicklaus Children's Hospital Genetics Clinic in Miami, Florida, for an evaluation, given symptoms of developmental delay and possible autism spectrum disorder. He was noted to have dysmorphic facial features, joint hypermobility, skin hyperpigmentation, nystagmus, as well as delayed and poor communication, including one-word utterances. Due to the dysmorphic facial features concerning for Noonan syndrome, a Noonan Syndrome and Related Conditions Panel (Laboratory Corporation of America Holdings (Labcorp), Burlington, North Carolina, United States) and a Chromosomal Microarray for Single Nucleotide Polymorphisms (Quest, Aliso Viejo, California, United States) were performed on a peripheral blood sample. The Noonan next-generation sequencing panel revealed that the patient was heterozygous for a variant of unknown significance in the lymphocytes (RAF1 c.1724 A>C missense variant). Additionally, the chromosomal microarray revealed a 1 Mb gain of 22q11.1q11.21 of undetermined significance, corresponding to a portion of the critical region for cat eye syndrome (CES). Because the patient did not meet the clinical criteria for CES, no further testing was performed. These genetic tests further demonstrate the difficulties of testing in mosaic conditions. Despite the incidental findings of unknown significance related to Noonan syndrome and CES, no definitive diagnosis was able to be made.

At three years of age, the child presented to a neurologist in Miami, Florida. A repeat brain MRI with contrast was performed, which revealed a 1.4 cm pineal lesion for which he was referred to neurosurgery. There was no associated hydrocephalus, and serum tumor markers were normal. Neurosurgery recommended a follow-up study with surveillance.

At four years of age, the patient underwent a repeat brain MRI, which revealed stability in the size of his pineal lesion. Serum tumor markers were again found to be normal. Exome sequencing was performed on a buccal swab and revealed a large multi-gene 12p13.33p11.1 duplication, present in 25-30% of sampled cells (GeneDx, Gaithersburg, Maryland, United States). This pathogenic CNV and the patient's clinical presentation were consistent with mosaic tetrasomy of chromosome 12p, indicating the presence of PKS mosaicism.

Table 1 summarizes the findings in this patient, classified by their embryological germ layer. This patient demonstrated symptoms that involved both ectoderm and mesoderm derivatives, and diagnosis was made by testing of epithelial cells from a buccal swab. These findings are further evaluated in the Discussion section, as they support the hypothesis that sampling from affected tissues may lead to a quicker diagnosis. 

**Table 1:** PKS GI: gastrointestinal; PKS: Pallister-Killian syndrome

Germ layer	Findings	Genetic testing
Ectoderm: epidermis, nervous system, salivary glands, buccal and anal openings, etc.	Global developmental delay, skin pigmentation changes, nystagmus, pineal mass, hypotonia	Buccal swab revealed a large multi-gene 12p13.33p11.1 duplication, present in 25-30% of sampled cells
Mesoderm: connective tissue, blood, muscle, kidneys, serous membranes, etc.	Joint hypermobility	Analysis of peripheral blood samples found no variants pertinent to the final diagnosis
Endoderm: lining of airways and parts of GI tract, digestive and endocrine glands, etc.	None	None

Case 2: TSC

A 32-year-old female initially presented at 30 years of age with a 25+-year history of facial angiofibromas and complaints of chronic pain and pressure when lying on her sides. Urology obtained ultrasound imaging of the patient's abdomen, which revealed multiple bilateral renal AML. The patient's urologist referred her to a geneticist, who ordered a TSC gene panel with next-generation sequencing and deletion-duplication testing (Invitae, San Francisco, California, United States). Analysis of TSC1 and TSC2 in a sample of the patient's saliva detected no pathogenic variants.

Approximately two years later, the patient presented to the clinic for preconception genetic counseling. A more comprehensive gene panel for TSC1 and TSC2 was carried out on samples of the patient's blood, saliva, skin, and AML core biopsies, using high-depth hybrid-capture next-generation sequencing (Illumina, San Diego, California, United States). No pathogenic variants in the TSC1 or TSC2 genes were detected in the blood or skin samples; however, the AML core biopsy samples revealed a nonsense mutation (TSC2: c.2251C>T) at high frequencies (41.3% and 72.8%), consistent with mosaicism for TSC. At this time, in contrast to previous testing, the variant was also found at a very low frequency (0.36%) in the sample of the patient's saliva.

Table 2 summarizes the findings in this patient, with symptoms involving tissues of mesodermal origin. With the biopsy of AML (mesoderm) yielding a TSC diagnosis, this highlights the potential clinical utility in targeted testing of affected tissue, or embryologic relatives, when initial genetic tests do not provide conclusive results. The idea of targeted testing for mosaic conditions is further analyzed in the Discussion section.

**Table 2:** TSC GI: gastrointestinal tract; AML: angiomyolipoma; TSC: tuberous sclerosis complex

Germ layer	Findings	Genetic testing
Ectoderm: epidermis, nervous system, salivary glands, buccal and anal openings, etc.	None	Analysis of TSC1 and TSC2 in samples of the patient's saliva detected a pathogenic allele in 0.36% of collected cells. Biopsy of the unaffected skin epithelium found no pathogenic variants
Mesoderm: connective tissue, blood, muscle, kidneys, serous membranes, etc.	Facial angiofibromas, bilateral renal AML	AML biopsies revealed a nonsense mutation at high frequencies (41.3% and 72.8%). Blood sample analysis was negative for pathogenic variants
Endoderm: lining of airways and parts of GI tract, digestive and endocrine glands, etc.	None	None

## Discussion

Mosaic conditions present a unique diagnostic complication. Given that the mutated cells are present at very low numbers or even absent in some tissues, genetic testing of standard tissue samples may not reveal the pathogenic variant. For example, a blood test may not reveal a mutation that could otherwise be found using a skin biopsy or buccal swab. Both the PKS and TSC cases presented demonstrate similar scenarios, where first-line testing failed to result in a diagnosis. This can have significant consequences for healthcare professionals and their patients, as the time to diagnosis is lengthened. A delay in diagnosis results in an inability to carry out targeted treatments, offer preventative care, and obtain insurance coverage for surveillance and management related to the diagnosis. Acquiring the correct tissue sample is critically important in diagnosing mosaic conditions. The absence of pathogenic variants in standard tissue samples is not sufficient to rule out mosaicism, as it does not exclude the possibility of the presence of the variant cell line in other tissue types. If initial testing is negative for a patient presenting with symptoms pathognomonic for a disease, reflex testing should include testing a sample of an affected tissue. Reflex testing is a second-line testing strategy intended to identify the pathogenic variant after the first-line test results are negative. This type of testing may involve testing for different genes, using a different testing technology, or testing a different patient sample. Testing standard unaffected tissue samples may fail to provide insight to the specific organ systems affected by a mosaic condition. An improved understanding of how to test for mosaic conditions is critically important for healthcare providers. Next, we discuss potential guidelines to better diagnose mosaic conditions. In some cases, invasive and potentially expensive biopsy may outweigh the benefits of a diagnosis. For example, if a patient is being managed symptomatically and a positive test result would not change treatment, biopsy may not be necessary. Shared decision-making should be used between clinicians and patients to determine if further testing is necessary.

When standard tissue samples yield negative results, physicians can reflex to testing a patient's tissues that display physical manifestations of their condition. As with cancer, a common type of mosaicism, the affected tissue is typically excised and biopsied to identify the pathogenic variant which will inform the best course of treatment, surveillance, and management.

As seen in Case 1: PKS, the 12p duplication characteristic of PKS was found in the patient's buccal swab, which is not surprising given the presence of hyperpigmentation changes in the epithelial cells of their skin. Theda et al. reported that buccal swab samples have a higher proportion of epithelial cells compared to saliva samples [15]. Results of Theda et al.'s study showed that both buccal and saliva samples contain leukocytes (mesoderm derivative) and epithelial cells (ectoderm derivative). The proportion of epithelial cells to leukocytes was highly variable in saliva samples; these inconsistencies in cell type and number across saliva samples can lead to variable results when testing for mosaic conditions. Buccal swabs, however, provide a greater quantity of epithelial cells for testing, providing for a higher chance of detecting a variant than a saliva sample. In this case specifically, the patient presented with symptoms manifested in ectoderm derivatives, so we would expect the epithelial cells to have a higher likelihood of showing the variant.

In Case 2: TSC, the pathogenic variant in the TSC2 gene was not detected until a sample of the patient's renal AML was tested. It is important to note, however, that biopsy of a patient's affected tissue may require an invasive procedure, which limits its clinical utility as a means of diagnosis. A patient's decision to undergo biopsy of an affected tissue may be impacted by the severity of their disease. In cases in which biopsy is feasible, it provides an alternate approach when initial standard testing fails to provide a diagnosis.

These cases both support the suggestion to sample affected tissues, or tissues from the same precursor germ layer, as it is likely to be a better diagnostic tool for mosaic diseases. In a study of MDS cases, Papavassiliou et al. found evidence supporting this idea [16]. When studying ectoderm-derived tissues, there was a correlation between IQ and buccal mucosa trisomy levels. Similarly, tissues of mesodermal origin demonstrated a correlation between congenital heart defects and lymphocyte mosaic trisomy levels. This evidence further supports our hypothesis that testing samples from affected tissues will provide for more timely diagnoses in mosaic conditions. An understanding of embryology allows one to predict which tissues will display the affected phenotype. This can help to shorten a patient's diagnostic odyssey, providing for earlier treatment of their condition and improved patient outcomes.

A pressing concern of patients with mosaic conditions is whether the mutation is present in the germline, as somatic and germline mosaic conditions have implications on family planning. This is exemplified by the patient with TSC, who sought genetic counseling to discuss the chance of passing on her TSC2 variant to her children. Understanding recurrence risk for patients with mosaic conditions is important in family planning. Studies have shown that undetected parental mosaicism could be a cause of what was originally believed to be de novo mutations in an embryo [17,18]. Specifically, two groups who performed genetic testing of paternal sperm and blood in unaffected parents found mosaic variants that led to intellectual disabilities and autism spectrum disorder in their offspring [17,18]. It is reasonable to assume that if an unaffected parent is able to pass down that variant to their child, the same could be said of an affected parent. There may be a higher recurrence risk from affected parents, due to a theoretically higher proportion of variant-harboring cells. Campbell et al. stated that parental somatic mosaicism increases the recurrence risk of transmission of variants [19]. Additionally and unsurprisingly, they found that if a patient harboring a mutation passes on the variant in their first pregnancy, they are more likely to do so in the following pregnancies, as the variant is present in the germline. Ye et al. suggested that recurrence risk in offspring may also be related to a patient's MAP, associating higher MAPs with a higher recurrence rate in postzygotic mosaicism [20]. As previously mentioned, the timing of the mutational mechanism determines what cell lines harbor the variant. Consequently, if a mutation occurs earlier in the development of a cell line and occurs in a cell line that will produce gametes, there will likely be a significantly higher recurrence rate. Both of these findings could be used to counsel patients about their recurrence risk.

Limitations of this case report include only reporting on two cases of mosaicism. Strengths include the novelty of testing affected tissues in mosaic conditions, as few publications currently exist on the topic. Additionally, the two cases presented above demonstrate our message of targeted testing in mosaic conditions despite the differences of timing of the mutations in relation to fertilization.

## Conclusions

Timely diagnosis of mosaic conditions is paramount for the treatment and management of disease and genetic counseling. Advancements in technology, decreased costs, and increased accessibility have made genetic testing easier than ever before. However, mosaicism does increase the difficulty of diagnosing patients, and sample choice should be considered when providers begin genetic testing. If a patient suffers from a disease that is genotypically mosaic, physicians should consider what tissues may be affected and use this as a guideline for diagnosis. Each of the two cases presented demonstrates how mosaicism can increase the length of a patient's diagnostic odyssey. When initial genetic testing results are negative and there are clinical findings consistent with genetic disease, physicians should refer patients to genetic specialists who can do a more intensive genetic testing protocol. A quicker diagnosis can lead to quicker treatments and better patient outcomes.
